# Supplementary material for: Emergent disorder and mechanical memory in periodic metamaterials
Source: Nat Commun. 2024 May 21;15:4008. doi: 10.1038/s41467-024-47780-w (PMC11109184; doi:10.1038/s41467-024-47780-w)
Supplement: Supplementary file 1 — Supplementary Information [file 41467_2024_47780_MOESM1_ESM.pdf]

Supplementary Information for:
Emergent Disorder and Mechanical Memory in Periodic Metamaterials

Chaviva Sirote-Katz, Dor Shohat, Carl Merrigan, Yoav Lahini, Cristiano Nisoli, and Yair Shokef

I. EXPERIMENTAL METHODS

To realize the frustrated Chaco lattice, we design a network of elastic beams, tracing the geometry of the Hookean springs in our theoretical model. Using a 3D printer (Prusa MK3 i3) we manufacture molds for the network, which are then used to cast samples made from silicone rubber (Mold Max™) with a Shore hardness of 30 A. The geometric dimensions of the rubber samples are shown in Fig. S1.

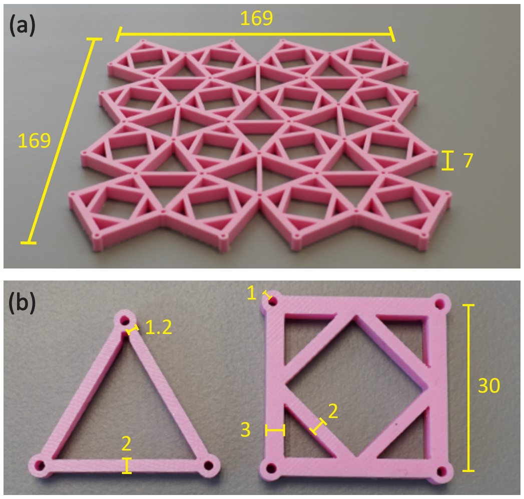

FIG. S1: Geometric dimensions (in mm) of the experimental Chaco lattice.

The basic beam length, constituting half the edge of each square or triangular unit in the Chaco lattice is equal to $\ell = 15mm$. To induce prestress in the samples, we constrain the elastic network to dimensions smaller than its rest configuration, using a set of pins, $2 \pm 0.01mm$ in diameter. Each pin acts as a rotation axis fixed in space. The spacing between neighboring pins is set to induce a uniform compression factor of $\alpha = 0.92 \pm 0.01$. To limit the deformation of the central beam between two triangles to its first buckling mode, we remove from the triangles the elastic beams which trace the k_2 springs. Instead, the region between squares meeting at a vertex of the lattice serve as a coupling mechanism between the deformation of adjacent beams in each triangle. The thickness of this region tunes the corresponding coupling k_2/k_1. The k_2 springs are not removed from the squares, however, to ensure the squares do not exhibit additional metastable states. Altogether, the geometry and α ensure that different beams do not form substantial contact, thus avoiding additional interactions or self-locking effects [1, 2].

The control sequence protocols are performed manually. The beams corresponding to the k_2 springs allow easy manipulation of the squares between their two stable states. We force the squares to flip by pushing one of their outer beams. See Supplementary Movie [3]. Experiments are documented using a digital camera (Sony alpha3). In Figs. 4, 5 and 6, the background is digitally removed for clarity.

II. COMPUTATIONAL METHODS

We simulate the harmonic springs theoretical model for the Chaco metamaterial with overdamped dynamics, or the method of steepest energy descent. The equation of motion for a given point with position $\vec{r}_i$ is given by

$$\frac{d\vec{r}_i}{dt} = \frac{1}{\gamma}\sum_{\langle ij\rangle} -k_{ij}(e_{ij} - l_{ij})\hat{r}_{ij}, \tag{1}$$

where the spring connecting point i to point j has spring constant k_{ij}, relaxed length l_{ij}, and current extension $e_{ij} = |\vec{r}_i - \vec{r}_j|$, and γ is a linear drag coefficient, which sets the relaxation time scale in the system, which, we set to $\gamma/k_1 = 1$. However all the results we present are for the final state after the system has fully relaxed, thus this time scale is not relevant for the results we present. Energy is minimized by integrating the equations of motion with variable time step $\Delta t = 0.01 - 0.1$.

For Figs. 3 and 4, we define the energy contained within each of the mechanical units, accounting for contributions from both k_1 and k_2 bonds, so that summing up all unit energies gives the total lattice energy. Each k_1 bond in the Chaco lattice is shared between two mechanical units, either between a square and a triangle or between two triangles. All the k_2 bonds are internal to a single unit. Thus we define the energy of each unit as the sum of harmonic spring energies of all its internal k_2 bonds plus one half the harmonic spring energy for each k_1 bond along the boundary of the unit.

III. MULTISTABILITY AND COLLAPSE TO THE GROUND STATE

Figure S2 compares the relative energy contributions from the square and triangular units as k_2/k_1 is gradually increased starting from four classes of initial configuration: the mechanical ground state [Fig. 3(a)], a configuration derived from a Shakti-lattice ASI ground state without paired defects [Fig. 3(b)], a state with randomly chosen relaxed orientations for all the squares [Fig. 3(c)], and finally a state with random displacements $\vec{s}_i(0)$ of all edges. Once the starting metastable state becomes unstable, some edges within the lattice flip, creating sharp drops in the energy.

In the mechanical ground state [Fig. 3(a)], energy is localized to the triangles, with full localization $E^{\Delta}/E_0 \to 1/2$ as $k_2/k_1 \to 0$. This behavior is also seen for the Shakti-lattice ASI ground state [Fig. 3(b)], which implies that the energy is also localized to the triangles, and further implies that the gap between the Shakti ASI ground state and the true mechanical ground state closes as $k_2/k_1 \to 0$. For larger k_2/k_1 the triangles and the squares in the Shakti ASI ground state have higher energy compared to the mechanical ground state. The energy of the squares in the Shakti ground state increases smoothly until

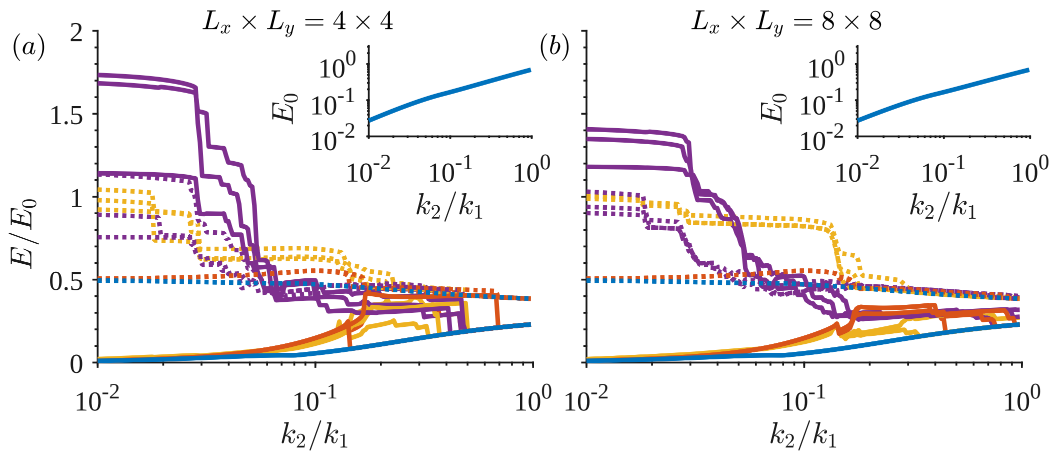

FIG. S2: Relative energy of the squares, $E^{\square}/E_0$ (solid) and of the triangles, E^{Δ}/E_0 (dotted), both normalized by the average unit energy in the ground state $E_0 = E^{\square} + 2E^{\Delta}$ (shown in the insets) as k_2/k_1 is slowly increased for systems of 4×4 (a) and 8×8 (b) squares with periodic boundary conditions. The line colors correspond to four classes of initial state: mechanical ground-state (blue), Shakti ASI ground-state with unpaired defects (red), randomly-oriented relaxed squares (yellow), and random displacements (purple). Three distinct initial conditions are shown for each class to illustrate the stochastic variations between runs. In (b), not all runs collapse to the ground state curves as k_2/k_1 increases because of competing ground-state domains in the larger system.

$k_2/k_1 \approx 0.15$, where sharp jumps start to appear, indicating that some squares flip so that excited triangles can become paired.

For relaxed squares in random orientations [Fig. 3(c)], the energy is also localized to the triangles, but the presence of higher-order excitations on the triangles causes the energy stored to be nearly twice that of the triangle energies in the mechanical ground state, giving $E^{\Delta}/E_0 \to 1$ as $k_2/k_1 \to 0$. Finally, the state with random edge displacements has large energy stored in both the squares and the triangles. As k_2/k_1 increases, edges become unstable and flip, taking the configuration towards the ordered ground state. The three metastable states shown all reach the ground state for $k_2/k_1 > 0.5$. In larger systems, the true ground state is not always fully reached because several domains with different ground-state orientations may form.

Figure S2 plots the relative energy curves for two system sizes and also shows three separate initial conditions for the different classes considered. Other than stochastic variations in the positions of the energy jumps, due to different initial conditions, the intensive relative energy curves show the same behaviors for each class. The important major difference that appears in the larger system is some of the energy curves do not collapse to merge with the mechanical ground state curves as k_2/k_1 increases. Inspection of the real space evolution of the configurations for the larger system in Fig. S3 shows that the failure to collapse completely to the ground-state curves is a result of the presence of multiple competing ground-state domains.

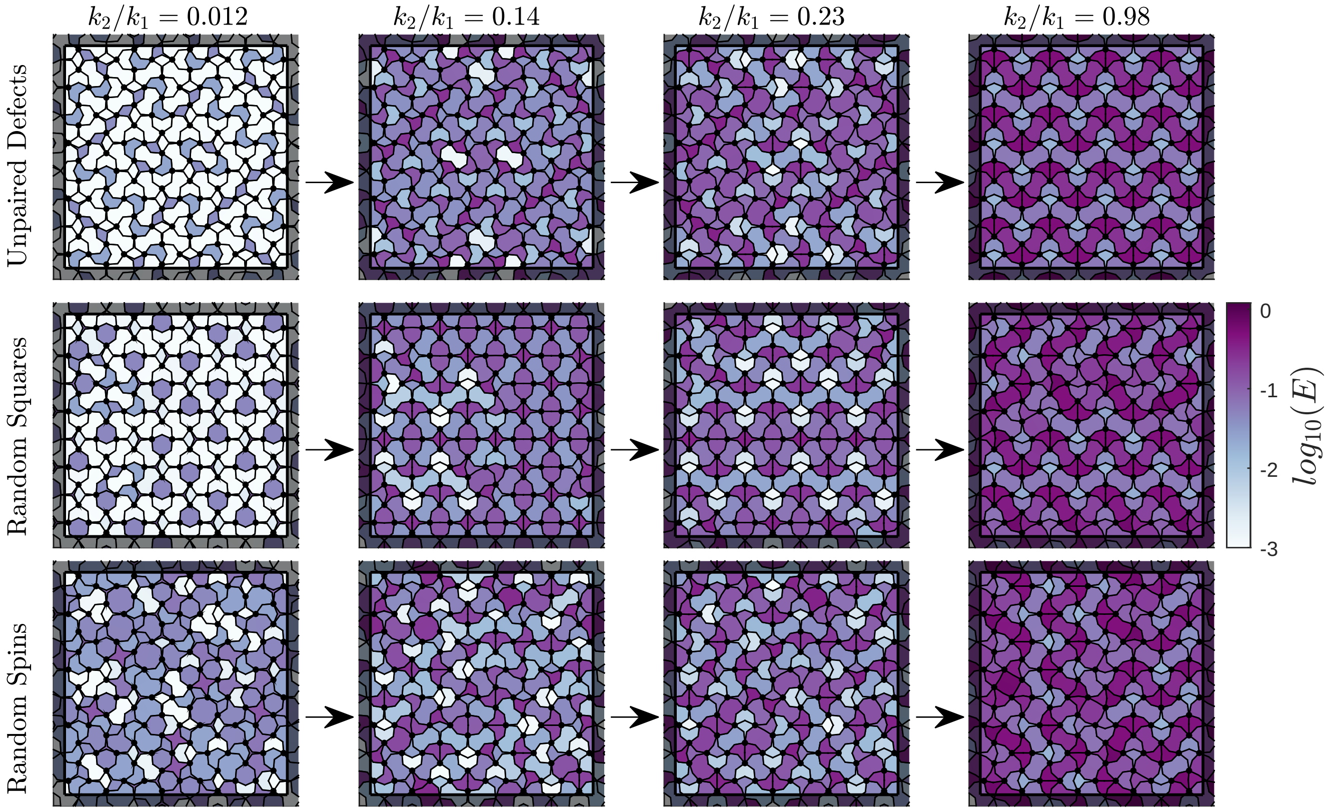

FIG. S3: Real space evolution of the metamaterial configurations for a system of 8×8 squares, for the three classes of initial condition, unpaired defects corresponding to a magnetic Shakti ground state, random initial choice of squares in their minimum energy orientations, and random choice of the edge displacements. As k_2/k_1 increases, edges inside the system start to flip to bring the configuration closer to the mechanical ground state. However, for the k_2/k_1 values shown here, there are still several competing ground-state domains, separated by domain walls.

[1] H. Fang, S. Li, and K. Wang, Self-locking degree-4 vertex origami structures, Proceedings of the Royal Society A: Mathematical, Physical and Engineering Sciences **472**, 20160682 (2016).

[2] H. Fang, S.-C. A. Chu, Y. Xia, and K.-W. Wang, Programmable self-locking origami mechanical metamaterials, Advanced Materials **30**, 1706311 (2018).

[3] See Supplemental Material at `https://youtu.be/9FKM54Lb5gU` for movie of non-Abelian response in experiments of the Chaco metamaterial undergoing the same operations at different sequences.
