# Supplementary material for: Emergent disorder and mechanical memory in periodic metamaterials
Source: Nat Commun. 2024 May 21;15:4008. doi: 10.1038/s41467-024-47780-w (PMC11109184; doi:10.1038/s41467-024-47780-w)
Supplement: Supplementary file 2 — Description of Additional Supplementary Files [file 41467_2024_47780_MOESM2_ESM.pdf]

Description of Additional Supplementary Files

File name: Supplementary movie 1.

Description: Non-Abelian response in experiments of the Chaco metamaterial undergoing the same operations at different sequences.
